# Developing a Measurement Scale of Gender-Friendly Hospital Environments: An Exploratory Study of Customer Perceptions in Taiwan

**DOI:** 10.3390/ijerph15102227

**Published:** 2018-10-11

**Authors:** Ying-Chyi Chou, Van Thac Dang, Hsin-Yi Yen, Pi-Shan Hsu

**Affiliations:** 1 Department of Business Administration, Tunghai University, Taichung 40704, Taiwan; ycchou@thu.edu.tw; 2 Business School, Research Institute for Guangdong-Taiwan Business Cooperation, Shantou University, Shantou 515063, China; 3 Department of International Business, Providence University, Taichung 43301, Taiwan; s8817007@mail2000.com.tw; 4 Department of Family Medicine, Taichung Hospital, Ministry of Health and Welfare, Taichung 40704, Taiwan; pshsu3@gmail.com

**Keywords:** healthcare environment, gender differences, customer perception, customer loyalty, customer willingness to pay

## Abstract

According to the United Nations, males and females should be given equal treatment in physical and psychological services, and healthcare institutions should exert greater efforts to reduce the gap in gender equality. However, this issue has been largely ignored in previous literature on healthcare environments. Designing a hospital environment that focuses on gender differences is critical to academic researchers and practical managers in all healthcare institutions. Thus, as an exploratory effort, this study aims to develop a measurement to assess customer perceptions of gender-friendly hospital environments. To identify and refine the structure of the instrument, two studies are conducted at different hospitals in Taiwan. The exploratory evidence shows there are five factors (i.e., physical design, functional design, marking design, gender perception, and gender-friendly services) and 28 items in the measurement scale of gender-friendly hospital environments. Results also show that gender-friendly hospital environments affect customers’ loyalty and willingness to pay. Based on our findings, hospital practitioners and researchers can adopt the measurement instrument used in this study to deal with the gap of gender equality in healthcare environments.

## 1. Introduction

A healthcare physical environment is a place where patients with health conditions go for treatment [1]. The quality of the healthcare physical environment has a significant influence on patients’ outcomes, and a good physical environment design helps promote the health and well-being of individual patients [2]. In recent years, the role of the healthcare physical environment has been a key focus in the holistic treatment of patients [3]. Healthcare environment designs have gradually shifted from emphasizing efficiency in terms of costs and clinical functionalities to customer-oriented designs that fit the demands and preferences of current and potential customers [4,5]. The movement toward customer-oriented designs requires hospitals to consider the holistic physical, psychological, and social well-being of its customers [3]. Despite this shift in hospital environment designs, as discussed in previous literature [1,3,6], the important role of gender differences in hospital environment designs has been largely ignored. The scant research attention directed to this topic provides a small number of references for theoretical researchers and hospital practitioners.

Healthcare patients have more options to choose from in a competitive market. Patients often make comparisons with other healthcare providers, and they are becoming increasingly aware of their central role in the type of healthcare they purchase [3]. Customer awareness and perceptions require healthcare providers to invest more resources and efforts to enhance their customers’ satisfaction [7]. Among the many factors that improve hospital quality, gender differences are important in the quality management system of a hospital [8]. Considering gender differences when designing hospital environments may be extremely useful for hospital architects, administrators, managers, and researchers of healthcare environments [9]. The purpose of this study is to develop a measurement scale for assessing customers’ perceptions of gender-friendly hospital environments. This measurement scale considers gender differences as the main attributes in designing a hospital environment. As a result, the proposed measurement scale will provide the first important step in the process of building and improving healthcare environments. This study provides foundations for hospital and environmental researchers interested in healthcare quality and for hospital administrators seeking ways to enhance the well-being and satisfaction of their customers.

The rest of this paper is structured as follows: Section 2 reviews previous literature and develops the hypotheses. Section 3 describes Study 1, which was designed to explore the constructs and items of the measurement scales of gender-friendly hospital environments. Section 4 describes Study 2, which was conducted to refine the final measurement scales and test the hypotheses. Finally, Section 5 concludes the paper with a discussion of the findings, and their implications.

## 2. Literature Review

### 2.1. Theoretical Basis for Gender-Friendly Hospital Environments

Traditional healthcare environment designs considered only the visions of architects, construction engineers, and administrators. This lack of customer-centered design has led many hospitals to build healthcare environments that primarily focus on efficiency and clinical functionality [4]. A research model of a “more humane hospital environment” [10] can be traced back to the hospital model of the Planetree philosophy [11,12]. This model emphasized healthcare settings with aesthetic, comfortable, soothing, and home-like environments [13]. Another notable model is the evidence-based design (EBD) [14], which is not only based on the designers’ technical knowledge and requirements, but also considers customers as the center of the design process [15]. The movement toward customer-oriented design has led to current research frameworks that relate hospital design attributes to the customers’ health and well-being [2,16,17]. A well-known model is the perceived hospital environment quality indicators (PHEQIs) [3,17,18], which focus on the physical and social environments of a healthcare setting from a customer-centered care perspective. The measurement scale of PHEQIs consist of four constructs, namely, external hospital spaces, hospital care unit, in-patient/waiting area, and social-functional features. One major advantage of this scale is its coverage of the main design attributes, which represent distinct features characterizing the physical and social aspects of hospital humanization [18]. The PHEQI scale also has good psychometric quality with high reliability and validity [3,17]. Unfortunately, none of the abovementioned research frameworks considers the role of gender differences in healthcare environment designs. Therefore, a measurement that assesses customer perceptions on gender-friendly hospital environments will be a valuable tool for architects, designers, administrators, and researchers in the field of healthcare environments.

The concept of gender mainstreaming, as proposed by the United Nations, has been widely discussed in the last decades. Mainstreaming involves ensuring that gender perspectives with the aim of gender equality are central in all research, legislations, and policies in the entire United Nations system. In hospital service environments, gender equity objectives have been identified in the position statements of the World Health Organization (WHO). The policy of gender equity is to reduce the health gaps between males and females, and enhance gender equality in health systems and healthcare services. Researchers have pointed out the differences between the healthcare service processes of men and women [9,19]. Doyal [20] suggested that men and women differ in physical, physiological, and psychological characteristics, and these gender differences may lead to gaps between their work environments, health conditions, and quality of life. Bean-Mayberry et al. [19] investigated the perceptions of female patients on the services provided by traditional healthcare centers and other gender-friendly healthcare centers. The authors indicated that female patients show a higher level of satisfaction toward the services of gender-friendly healthcare centers than do male patients. Huisman et al. [1] also suggested that healthcare environments should consider gender differences in the healthcare service process, and hospital environment designs should consider the different demands regarding motivation, privacy, value, and self-respect between men and women.

Combining the concept of gender differences with hospital environment design is a good strategy to enhance the quality of hospital healthcare services and increase gender equality in health systems and healthcare services [21,22]. From a customer-centered perspective, physical and social factors should be integrated into the measurement to develop a psychologically sound scale of gender-friendly hospital environments [3,23]. Bitner [24,25] suggested three important dimensions of the physical environment, namely, surrounding environments, spaces/functions and physical signs, and symbols and images. In addition, Ulrich et al. [2] indicated the importance of social interactions between hospital staff members and patients in the social environment of healthcare services. Therefore, based on previous literature on healthcare environments, the domain and scope of the measurement of a gender-friendly hospital environment should include physical and social environments [2,3,11,13,14,16,17,18]. Specifically, measurements of the physical environment reflect the quality of the surrounding environments (e.g., temperature, air quality, sound, and colors), spaces/functions (e.g., facilities, spatial comfort, spatial configuration, and view planning), and physical signs, symbols, and images [3,18,25]. Moreover, measurements of the social environment indicate the service attitudes (e.g., the hospital staff’s awareness and attitudes about gender differences) and service behaviors (e.g., the hospital staff’s behavioral interactions with patients regarding gender difference services) [2,21,22].

### 2.2. Effects of Gender-Friendly Hospital Environments on Patient Outcomes

Several studies have investigated the impact of the quality of a hospital’s environment on patient outcomes, including health recovery, hospital perception, satisfaction, and overall well-being [3]. Arneill and Devlin [16] showed that a hospital’s physical environment influences the perceptions of patients on the quality of care. Patients were satisfied with waiting rooms that were nicely furnished, well-lit, contained artwork, and were warm in appearance. Leather et al. [26] found that a hospital’s environmental attributes are associated with improved mood, altered physiological states, and high levels of satisfaction. Swan, Richarson, and Hutton [27] also demonstrated the relevance of a positive patient evaluation of hospital patient rooms that were hotel-like and were well-decorated with art, carpeted floors, wooden furniture, crown molding, and ceramic bath tiles. Furthermore, in a paper on hospital environment issues, Ulrich et al. [2] summarized the relationships between several environmental design factors and patients’ healthcare outcomes. The authors concluded that physical environmental factors (e.g., designs of patient rooms, lighting, color, noise, temperature, and flooring) have direct and indirect influences on patients’ health conditions and well-being (e.g., depression, stress, sleep patterns, pain, length of stay, health recovery, satisfaction, and many other factors). These findings suggest that patients are aware of the quality of the hospital environment and that these perceived qualities have an impact on their outcomes [3].

Eliminating gender disparities is an important issue that has been proposed by the United Nations. According to the WHO, reducing the gap in gender equality should be addressed in all healthcare institutions. Accordingly, the concept of gender-friendliness in hospital environments deals with the differences between male and female patients [9,19]. Healthcare institutions should design environments that care about gender differences and provide equal services for male and female patients [20]. A physical and psychological hospital environment that is gender-friendly and comfortable will help enhance the well-being of male and female patients [20].

From the lack of literature on gender-friendly hospital environments, we have drawn theoretical foundations from literature on environmental psychology and marketing. Stimulus-organism-response (S-O-R) theory is an appropriate framework to interpret the influences of gender-friendly hospital environments on patient outcomes. The S-O-R model is an extension of the classic stimuli-response theory that integrates the role of an organism into the link between stimuli and response. The theory posits that environmental cues (stimuli) influence an individual’s internal evaluation (organism), which further produces behavioral outcomes (response) [28,29]. When the S-O-R model is applied to the context of gender-friendly hospital environments, the environmental stimuli are the various physical factors and social factors, such as surrounding environmental attributes, physical signs, symbols, and images, as well as the attitudes and behavioral services of the hospital staff regarding gender-friendly issues. When customers are exposed to and interact with the hospital environment, the stimuli will affect their perceptions and evaluations, which produce reactions. The customers’ reactions are likely to shape their purchase and post-purchase behaviors, such as loyalty and willingness to pay [28,30,31]. Therefore, on the basis of the aforementioned literature and the logic of the S-O-R theory, we argue that a hospital with a gender-friendly environment will influence patient outcomes in a positive manner. The following hypotheses are thus presented:

**Hypothesis** **1.** *Gender-friendly hospital environments will be positively related to customer loyalty.*

**Hypothesis** **2.** *Gender-friendly hospital environments will be positively related to customer willingness to pay.*

## 3. Study 1

### 3.1. Sample and Procedure

The purpose of Study 1 is to identify the constructs of the measurement scale of gender-friendly hospital environments. Initial items for gender-friendly hospital environments were generated from two sources. First, we selected key words regarding gender differences based on a literature review of hospital environment quality and environmental psychology [2,3,17,18,32,33,34]; and second, a brainstorming session with experts and doctors was used to generate and refine the measurement items. Modifications on the wording and depletion of the items based on a pilot test were carried out in the first phase of the data collection procedure. Items with unclear, overlapping, or similar meanings, which may cause misunderstandings, were deleted. The remaining 29-item preliminary measure was used to construct a formal questionnaire in the second phase of the data collection procedure. The questionnaires were delivered to patients in hospitals in central Taiwan in 2016. Participants were asked to complete the questionnaire with a five-point Likert-type scale ranging from 1 (strongly disagree) to 5 (strongly agree). A final sample of 203 completed questionnaires was returned, which was used for the exploratory factory analysis (EFA). Table 1 shows the demographic profiles of the respondents.

### 3.2. Results of the EFA

To determine the factorial structure of the measurement scale of gender-friendly hospital environments, an EFA was applied using a maximum likelihood method with a varimax rotation. According to the EFA method, the threshold values of factor loadings for the items are greater or equal to 0.5, and Cronbach’s coefficient alpha for multi-item measures are acceptable at 0.6 or above [35,36,37]. The results of the EFA are presented in Table 2. As shown at the bottom part of Table 2, the Kaiser-Meyer-Olkin (KMO) sampling adequacy measure was 0.930, and Bartlett’s test of sphericity, which tests whether the correlation matrix is an identity matrix, was significant (*p* < 0.001). This finding indicates an acceptable level of factorability for the factor analysis. Furthermore, a total of five factors were identified with an eigenvalue greater than unity, which cumulatively explains 79.51% of the variance. All factor loadings of items on each factor were greater than 0.5, except one item with cross loadings. A detailed look at the factor loadings of these five factors indicates that the first factor of gender-friendly hospital environments consisted of five items that measured the surrounding physical environments of a hospital—thus, it was named “physical design”. The second factor consisted of seven items (except for one item with cross loadings, which was deleted) that measured the space and functional design of a hospital and was thus named “functional design”. The third factor consisted of five items that measured the physical signs, symbols, and image design of a hospital and was thus named “marking design”. The fourth factor consisted of eight items that measured the awareness and attitude of a hospital on gender-friendly issues; thus, it was named “gender perception”. The final factor consisted of three items that measured the gender-friendly service quality of a hospital and was thus named “gender-friendly services”. The average loading of the 28 items on their respective factors of gender-friendly hospital environments was 0.75. The internal consistency reliability for physical design, functional design, marking design, gender perception, and gender-friendly services were 0.94, 0.95, 0.92, 0.95, and 0.91, respectively. These results indicate good reliability of the measurement scale. Therefore, the final constructs and items for the measurement of gender-friendly hospital environments consisted of five factors with 28 items, as shown in Table 2.

## 4. Study 2

### 4.1. Sample and Procedure

Although the results of Study 1 show the constructs and items for the measurement scale of gender-friendly hospital environments, stronger evidence may be needed to assure the structure of the instrument. The purpose of Study 2 is to refine the measurement scale generated in Study 1 and test the hypotheses of this study. A formal survey was conducted in hospitals in central Taiwan in 2017. In this study, the survey questionnaire included 28 items from the measurement generated in Study 1, and 10 items assessing the loyalty and willingness to pay of customers. Participants were asked to complete the structured questionnaire with a five-point Likert-type scale ranging from 1 (strongly disagree) to 5 (strongly agree). A final sample of 300 questionnaires were returned, of which 249 were valid, resulting in a response rate of 83%. Table 3 shows the demographic profiles of the respondents.

### 4.2. Measurements

#### 4.2.1. Gender-Friendly Hospital Environments

Twenty-eight items from the five factors of the measurement scale generated in Study 1 were used in Study 2. Physical design was measured with five items, functional design was measured with seven items, marking design was measured with five items, gender perception was measured with eight items, and gender-friendly services were measured with three items.

#### 4.2.2. Customer Loyalty

Customer loyalty is defined as a deeply held commitment to re-purchase a preferred product or service repetitively from the same brand, despite external influent factors to cause switching behavior [38]. To measure customer loyalty in the context of hospital environments, six items were revised from previous literature [31,38], with one example being, “This hospital will be my first choice in the future”.

#### 4.2.3. Customer Willingness to Pay

Willingness to pay is a measure of the value that a customer assigns to his/her consumption in monetary units. It is defined as the amount of money or price a customer is willing to pay for a product or service [39]. In the present study, four items were refined from previous literature to measure customer willingness to pay [30,40]. One example is, “I’m willing to pay a high price for services provided by this hospital”.

#### 4.2.4. Control Variables

Several demographic characteristics of the respondents were controlled in our analysis. Gender was coded with a dummy variable, where 0 indicates female and 1 indicates male; education was measured using five categories (0 = elementary school or below, 1 = junior high school, 2 = senior high school, 3 = undergraduate, and 4 = graduate or above); age was measured based on seven categories (0 = 19 years old or below, 1 = 20–29 years old, 2 = 30–39 years old, 3 = 40–49 years old, 4 = 50–59 years old, 5 = 60–69 years old, and 6 = 70 years old or above); and monthly income was measured using five categories (0 = below NT$30,000 (NT, Taiwan new dollar), 1 = NT$30,000–below NT$40,000, 2 = NT$40,000–below NT$50,000, 3 = NT$50,000–below NT$60,000, and 4 = NT$60,000 or above).

### 4.3. Empirical Results

#### 4.3.1. EFA

To refine the factorial structure of the measurement scale of gender-friendly hospital environments, an EFA was also applied in Study 2 by using a maximum likelihood method with a varimax rotation. The results of the EFA are presented in Table 4. As shown at the bottom part of Table 4, the KMO sampling adequacy measure was 0.947 and Bartlett’s test of sphericity was significant (*p* < 0.001). This result indicates an acceptable level of factorability for the factor analysis. It likewise shows that seven factors were identified with an eigenvalue greater than unity, and these seven factors cumulatively explained a total of 79.35% of the variance. All factor loadings of items on each factor were greater than 0.5. Customer loyalty consisted of six items, customer willingness to pay consisted of four items, and the remaining five factors represented the five dimensions of gender-friendly hospital environments. The first factor of gender-friendly hospital environments was physical design, which consisted of five items; the second factor was functional design, which had seven items; the third factor was marking design, which consisted of five items; the fourth factor was gender perception, which consisted of eight items; and the final factor was gender-friendly services, which consisted of three items. The average loading of the 28 items on their respective factors of a gender-friendly hospital environment was 0.69, and cross loadings were negligible. The values of internal consistency reliability for customer loyalty, customer willingness to pay, physical design, functional design, marking design, gender perception, and gender-friendly services were 0.95, 0.83, 0.93, 0.95, 0.94, 0.96, and 0.92, respectively. These results indicate good reliability of the measurement scale. The results of the EFA are shown in Table 4, and the details of each item of the gender-friendly hospital environment is presented in Appendix A.

#### 4.3.2. Common Method Variance Testing

Podsakoff, MacKenzie, and Lee [41] suggested that common method variance appears if the results of the principal component analysis show a single factor emerging from unrotated factor solutions, or a first factor explains the majority of the variance. Therefore, to detect possible common method variance, Harmar’s one-factor test was conducted by entering all items together into a principal component analysis [41]. The results indicated that no single factor emerged and that the first factor cannot explain the majority of the variance. Thus, common method variance did not seem to be a problem in our sample data.

#### 4.3.3. Descriptive Statistics

Table 5 shows the means, standard deviations, and Pearson correlations among the variables in this study. As shown in Table 5, the five dimensions of gender-friendly hospital environments were significantly and positively correlated to customer loyalty (physical design: *r* = 0.32, *p* < 0.01; functional design: *r* = 0.41, *p* < 0.01; marking design: *r* = 0.44, *p* < 0.01; gender perception: *r* = 0.35, *p* < 0.01; and gender-friendly services: *r* = 0.38, *p* < 0.01). In addition, the five dimensions of gender-friendly hospital environments were significantly and positively correlated to customer willingness to pay (physical design: *r* = 0.30, *p* < 0.01; functional design: *r* = 0.30, *p* < 0.01; marking design: *r* = 0.32, *p* < 0.01; gender perception: *r* = 0.33, *p* < 0.01; and gender-friendly services: *r* = 0.26, *p* < 0.01). Regression analysis was used to test the hypotheses in the next section.

#### 4.3.4. Hypothesis Testing

Table 6 presents the results of the effects of gender-friendly hospital environments on customer loyalty. As indicated in Table 6, control variables were entered into model 1a. The effects of each dimension and overall construct of gender-friendly hospital environments on customer loyalty were tested from models 2a to 7a. The results show that physical design was significantly and positively related to customer loyalty (model 2a: β = 0.53, *p* < 0.001), functional design was significantly and positively related to customer loyalty (model 3a: β = 0.50, *p* < 0.001), marking design was significantly and positively related to customer loyalty (model 4a: β = 0.49, *p* < 0.001), gender perception was significantly and positively related to customer loyalty (model 5a: β = 0.55, *p* < 0.001), and gender-friendly services were significantly and positively related to customer loyalty (model 6a: β = 0.38, *p* < 0.001). The overall results of the construct of gender-friendly hospital environments show that they were significantly and positively related to customer loyalty (model 7a: β = 0.58, *p* < 0.001). Thus, Hypothesis 1 was supported.

Table 7 shows the effects of gender-friendly hospital environments on customer willingness to pay. As indicated in Table 7, model 1b presents the effects of control variables. Models 2b to 7b present the effects of each dimension and the overall construct of gender-friendly hospital environments on customer willingness to pay. The results show that physical design was significantly and positively related to customer willingness to pay (model 2b: β = 0.31, *p* < 0.001), functional design was significantly and positively related to customer willingness to pay (model 3b: β = 0.33, *p* < 0.001), marking design was significantly and positively related to customer willingness to pay (model 4b: β = 0.34, *p* < 0.001), gender perception was significantly and positively related to customer willingness to pay (model 5b: β = 0.35, *p* < 0.001), and gender-friendly services were significantly and positively related to customer willingness to pay (model 6b: β = 0.27, *p* < 0.001). The overall results of the construct of gender-friendly hospital environments show that they were significantly and positively related to customer willingness to pay (model 7a: β = 0.36, *p* < 0.001). Thus, Hypothesis 2 was also supported.

## 5. Discussion and Conclusions

The purpose of this study was to develop an instrument to measure customer perceptions on gender-friendly hospital environments. As an exploratory effort, the results of Study 1 identified five factors, with 28 items in the instrument of gender-friendly hospital environments. The results of Study 2 refined the measurement scale and showed that the instrument consisted of five factors with 28 items, namely, physical design with five items, functional design with seven items, marking design with five items, gender perception with eight items, and gender-friendly services with three items. The empirical results of Study 2 also indicated the positive impacts of gender-friendly hospitals on customers’ loyalty and willingness to pay. The findings of this study provide important implications for academic researchers and practical managers.

### 5.1. Theoretical and Managerial Implications

From a research perspective, healthcare environment design is an important component in the healthcare quality system. In recent years, researchers have focused their attentions on customer-centered design; however, the role of gender differences has not been discussed in previous literature on healthcare environment design. This study integrated the concept of gender differences in hospital environment design, and thus contributes to the knowledge in the field of healthcare environments. In addition, it plays an important role as a first effort in developing an instrument for gender-friendly hospital environments, thus providing insights for understanding the critical role of gender differences and helping increase gender equality in health systems and healthcare services.

From a managerial perspective, based on the findings of this study, architects, designers, and planers can take advantage of a measurement that assesses the perceptions of customers on the quality of the healthcare environment. Hospitals should make changes toward customized services that consider gender differences as the main attributes in designing healthcare environments. In particular, hospitals should stress the attributes of physical environments, functionality, and marking designs, and these attributes should fit well with the demands of males and females. Furthermore, with respect to the social environment, hospitals should emphasize the importance of gender perceptions, as well as gender-friendly services. The findings of this study show that gender-friendly hospital environments have a positive influence on patient outcomes. This result provides implications for hospital administrators and managers in planning service strategies that reduce gender differences and enhance gender equality in hospital healthcare services. Such strategies reflect the needs and expectations of individual customers, thus increasing the customers’ loyalty and willingness to pay.

In summary, the attempt to develop an instrument for gender-friendly hospital environments in this study is the first important step in the research on healthcare environment design. This preliminary foundation contributes to the literature on hospital quality. This study also provides implications for hospital architects, designers, administrators, and managers in improving hospital service quality and enhancing the quality of health and well-being of customers.

### 5.2. Limitations and Directions for Future Research

Although this study has been designed meticulously, some limitations that should be addressed in future research are acknowledged. First, this study only identified and refined the factorial structure and items of the measurement scale of gender-friendly hospital environments. As an exploratory study, the validity of the instrument has not been tested in this study. Thus, additional evidence on the validity of the measurement instrument should be examined and confirmed in future research. Second, the measurement items were developed based on Taiwanese respondents and are thus only suitable for hospital environments in Taiwan. Future research can rely on our measurement instrument to examine the issue of gender-friendly hospital environments in other contexts. Third, the study deals only with two variables of customer outcomes, namely, loyalty and willingness to pay. Other possible outcome variables can include customer satisfaction, word of mouth, trust, and quality of healthcare treatment. These variables should be further examined in future research. Finally, patients are believed to be more sensitive to hospital environment designs regarding gender differences, because they are the central focus of the healthcare process. Thus, the measurement scale of this study was developed primarily to assess customer perceptions. Future research can consider the experiences of hospital staff members and the evaluations of hospital designers in designing the measurement instrument of gender-friendly hospital environments.

## Figures and Tables

**Table 1:** Demographic profiles of respondents in Study 1 (*n* = 203).

Variable	Frequency	Percent
Gender		
Male	115	56.70%
Female	88	43.30%
Education		
Elementary school or below	2	1.00%
Junior high school	2	1.00%
Senior high school	23	11.30%
Undergraduate	137	67.50%
Gradate or above	39	19.20%
Age		
19 years old or below	2	2.00%
20–29 years old	14	6.90%
30–39 years old	59	29.10%
40–49 years old	3	1.50%
50–59 years old	66	32.50%
60–69 years old	47	23.20%
70 years old or above	10	4.80%
Income		
Below NT$30,000	31	15.30%
NT$30,000–below NT$40,000	42	20.70%
NT$40,000–below NT$50,000	33	16.30%
NT$50,000–below NT$60,000	39	19.30%
NT$60,000 or above	58	28.40%

Note: NT = Taiwan new dollar.

**Table 2:** Results of the constructs and items of the measurement scale (Study 1, *n* = 203).

Constructs	Items	Factor Loadings	Eigenvalues	% of Variance	Cumulative %	Cronbach’s Alpha
Physical design (PD)	PD1	0.819	15.87	20.79	20.79	0.94
	PD2	0.861				
	PD3	0.838				
	PD4	0.847				
	PD5	0.825				
Functional design (FD)	FD1	0.754	2.83	18.99	39.79	0.95
	FD2	0.699				
	FD3	0.827				
	FD4	0.827				
	FD5	0.754				
	FD6	0.677				
	FD7	0.699				
Marking design (MD)	MD1	0.660	1.83	15.47	55.25	0.92
	MD2	0.626				
	MD3	0.647				
	MD4	0.712				
	MD5	0.688				
Gender perception (GP)	GP1	0.767	1.44	12.34	67.59	0.95
	GP2	0.727				
	GP3	0.772				
	GP4	0.623				
	GP5	0.880				
	GP6	0.757				
	GP7	0.665				
	GP8	0.793				
Gender-friendly services (GFS)	GFS1	0.833	1.09	11.92	79.51	0.91
	GFS2	0.788				
	GFS3	0.670				
KMO and Bartlett’s Test	KMO-Measure of Sampling Adequacy = 0.930 Bartlett’s Test of Sphericity: Sig. = 0.000					

Note: *n* = 203, varimax with Kaiser normalization, and rotation converged in 8 iterations. KMO, Kaiser-Meyer-Olkin.

**Table 3:** Demographic profiles of respondents in Study 2 (*n* = 249).

Variable	Frequency	Percent
Gender		
Male	111	44.60%
Female	138	55.40%
Education		
Elementary school or below	3	1.20%
Junior high school	6	2.40%
Senior high school	30	12.00%
Undergraduate	167	67.10%
Gradate or above	43	17.30%
Age		
19 years old or below	5	2.00%
20–29 years old	20	8.00%
30–39 years old	67	26.90%
40–49 years old	83	33.30%
50–59 years old	60	24.10%
60–69 years old	12	4.80%
70 years old or above	2	0.80%
Income		
Below NT$30,000	43	17.20%
NT$30,000–below NT$40,000	52	20.90%
NT$40,000–below NT$50,000	40	16.10%
NT$50,000–below NT$60,000	47	18.90%
NT$60,000 or above	67	26.90%

Note: NT = Taiwan new dollar.

**Table 4:** Results of the constructs and items of the measurement scale (Study 2, *n* = 249)

Constructs	Items	Factor Loadings	Eigenvalues	% of Variance	Cumulative %	Cronbach’s Alpha
Customer loyalty (CL)	CL1	0.799	20.80	14.81	14.81	0.95
	CL2	0.843				
	CL3	0.832				
	CL4	0.846				
	CL5	0.842				
	CL6	0.769				
Customer willingness to pay (WP)	WP1	0.861	3.73	14.67	29.48	0.83
	WP2	0.859				
	WP3	0.608				
	WP4	0.577				
Physical design (PD)	PD1	0.656	1.84	14.34	43.82	0.93
	PD2	0.673				
	PD3	0.667				
	PD4	0.719				
	PD5	0.726				
Functional design (FD)	FD1	0.697	1.52	10.33	54.15	0.95
	FD2	0.758				
	FD3	0.783				
	FD4	0.642				
	FD5	0.639				
	FD6	0.653				
	FD7	0.666				
Marking design (MD)	MD1	0.630	1.45	9.74	63.89	0.94
	MD2	0.719				
	MD3	0.778				
	MD4	0.719				
	MD5	0.723				
Gender perception (GP)	GP1	0.706	1.30	9.25	73.14	0.96
	GP2	0.627				
	GP3	0.696				
	GP4	0.623				
	GP5	0.796				
	GP6	0.546				
	GP7	0.563				
	GP8	0.729				
Gender-friendly services (GFS)	GFS1	0.785	1.10	6.22	79.35	0.92
	GFS2	0.737				
	GFS3	0.696				
KMO and Bartlett’s Test	KMO-Measure of Sampling Adequacy = 0.947 Bartlett’s Test of Sphericity: Significant = 0.000					

Note: *n* = 249, varimax with Kaiser normalization, and rotation converged in 8 iterations.

**Table 5:** Means, standard deviations, and Pearson correlations.

Constructs	Mean	S.D.	1	2	3	4	5	6	7
1. Customer loyalty	4.20	0.55	0.1						
2. Customer willingness to pay	3.72	0.60	0.44 **	1					
3. Physical design	4.43	0.46	0.32 **	0.30 **	1				
4. Functional design	4.33	0.54	0.41 **	0.30 **	0.41 **	1			
5. Marking design	4.25	0.53	0.44 **	0.32 **	0.28 **	0.37 **	1		
6. Gender perception	4.44	0.49	0.35 **	0.33**	0.35 **	0.37 **	0.35 **	1	
7. Gender-friendly services	4.25	0.65	0.38 **	0.26 **	0.36 **	0.37 **	0.32 **	0.30 **	1

Note: *n* = 249, ** *p* < 0.01.

**Table 6:** Regression analysis for the effects of gender-friendly hospital environments on customer loyalty.

Independent Variables	Dependent Variable: Customer Loyalty						
	Model 1a	Model 2a	Model 3a	Model 4a	Model 5a	Model 6a	Model 7a
Control variables							
Gender	−0.09	0.01	0.03	−0.01	0.05	−0.03	0.04
Education	−0.08	−0.07	−0.06	−0.07	−0.09	−0.06	−0.07
Age	−0.01	0.05	0.05	0.03	0.04	0.01	0.06
Income	0.13	0.07	0.11	0.12	0.05	0.13	0.09
Independent variables							
Physical design		0.53 ***					
Functional design			0.50 ***				
Marking design				0.49 ***			
Gender perception					0.55 ***		
Gender-friendly services						0.38 ***	
Overall gender-friendly hospital environments							0.58 ***
R^2^	0.03	0.30 ***	0.26 ***	0.25 ***	0.28 ***	0.15 ***	0.33 ***
ΔR^2^		0.27 ***	0.23 ***	0.22 ***	0.25 ***	0.12 ***	0.30 ***

Note: *n* = 249, *** *p* < 0.001.

**Table 7:** Regression analysis for the effects of gender-friendly hospital environments on customer willingness to pay.

Independent Variables	Dependent Variable: Customer Willingness to Pay						
	Model 1b	Model 2b	Model 3b	Model 4b	Model 5b	Model 6b	Model 7b
Control variables							
Gender	−0.04	0.03	0.05	0.02	0.06	0.01	0.06
Education	−0.06	−0.06	−0.05	−0.06	−0.07	−0.06	−0.06
Age	0.04	0.11	0.11	0.10	0.11	0.09	0.12
Income	0.02	0.03	0.05	0.06	0.01	0.06	0.04
Independent variables							
Physical design		0.31 ***					
Functional design			0.33 ***				
Marking design				0.34 ***			
Gender perception					0.35 ***		
Gender-friendly services						0.27 ***	
Overall gender-friendly hospital environments							0.36 ***
R^2^	0.02	0.11 ***	0.12 ***	0.13 ***	0.13 ***	0.09 ***	0.15 ***
ΔR^2^		0.08 ***	0.10 ***	0.11 ***	0.11 ***	0.07 ***	0.13 ***

Note: *n* = 249, *** *p* < 0.001.

## Appendix A. Measurement Scale of Gender-Friendly Hospital Environments

Physical design

1. The rooms are maintained at comfortable temperatures.
2. Air quality is good and there are no bad or pungent odors.
3. There are no disturbing noises in the surrounding environment.
4. The brightness of the lights is toned down to a comfortable level.
5. The design of the waiting areas meets the need for privacy.

Functional design

1. The spaces in the hospital are arranged in accordance with the need for privacy (i.e., there are individual check-up spaces with examination beds, screens, and blankets).
2. There are individual changing spaces (clean and with adequate covering).
3. Lactation rooms are clearly indicated throughout the hospital.
4. Lactation rooms are designed to provide adequate covering functions (e.g., chairs with back rests, trash cans with lids, power supplies, doors that can be locked from the inside, and washing facilities) and are equipped with alarms or other kinds of emergency systems.
5. Lavatories are equipped with alarms, hooks, and toilets, meeting the requirements for barrier-free environments, and hygiene items are provided.
6. There are family-friendly lavatories.
7. Facilities and procedures of facility usage are convenient for males and females.

Marking design

1. Gender-friendly signs (e.g., priority seat signs and family-friendly parking space signs) are provided.
2. Signs, bulletins, and promotional posters in the hospital can help increase gender-friendly awareness.
3. The healthcare center provides vests or blankets that prevent unintended bodily exposure.
4. Changing rooms are adequately sized.
5. Storage rooms are adequately sized and well designed.

Gender perception

1. The service staff are mindful of protecting patients’ privacy.
2. Check-ups are conducted in a professional manner, and there is no playful attitude or willful touching or spying on patients’ bodies.
3. Patients do not feel uneasy or embarrassed when facing staff members of the opposite sex.
4. Staff members possess the knowledge necessary to determine menstrual illness.
5. Staff members practice gender sensitivity when interacting with patients and will not cause patients discomfort during a treatment process.
6. If bodily exposure is required in tests, service staff members will actively provide adequate coverings or close screens.
7. Professional staff members will accompany patients during body check-ups and provide adequate assistance.
8. Non-medical staff members (e.g., intern physicians and trainees) can be present during the treatment process only with the patients’ permissions.

Gender-friendly services

1. The hospital has a sexual harassment prevention policy.
2. Gender differences are taken into consideration in designing the process of body check-ups and treatments (e.g., male and female patients are divided into two check-up lines so they will not be affected by other patients of the opposite sex).
3. There is a safety cab hailing service for female patients (e.g., hospitals write down the taxi driver’s administrative and license plate numbers when patients leave).
